# Treatment with the herbal formulation Eefooton slows the progression of chronic kidney disease: A case report

**DOI:** 10.1097/MD.0000000000017573

**Published:** 2019-10-25

**Authors:** Chien-An Yao, Chih-Hui Lin

**Affiliations:** a Department of Family Medicine, National Taiwan University Hospital, Taipei City; b Gerent Biotech R&D Center No. 20, Jen Yi St., Taichung City, Taiwan (R.O.C.).

**Keywords:** chronic kidney disease, end-stage renal disease, hemodialysis, herbal formula, peritoneal dialysis

## Abstract

**Rationale:**

Patients with end-stage kidney disease (ESKD) receiving maintenance dialysis experience an overall burden of physical and emotional symptoms. However, there were limited alternative treatments to dialysis.

**Patient concerns:**

A 79-year-old woman with chronic kidney disease stage 5 (CKD5) and gout had refused to be on dialysis. She also had hypoglycemia, hypertension, and heart disease.

**Diagnoses:**

The patient had received the ultrasonography, the renal biopsy and biochemical examinations, confirming the diagnosis of renal impairment, primary hypertension, and chronic nephritic syndrome with unspecified morphologic changes.

**Interventions:**

She was administered with 20 mL Eefooton (a liquid formula of herbal extracts: *Astragalus membranaceus* 3 g, *Codonopsis pilosula* 3 g, *Ligustrum lucidum* 3 g, *Panax quinquefolius* 1.3 g, and *Rhodiola sacra* 1.3 g) orally twice a day for 6 months in addition to her regular medications.

**Outcomes:**

The patient was followed up for 3 months after the completion of the Eefooton adjuvant treatment. The patient's renal function was improved, and CKD progression was alleviated. After Eefooton treatment, the sizes of both kidneys in the patient increased by 8% while blood urea nitrogen (BUN) and serum creatinine concentrations were decreased. In addition, further reduction in BUN concentration was observed 2 months posttreatment.

**Lessons:**

This case demonstrated that Eefooton has potential therapeutic significance in patients with CKD5 who chose conservative treatment over dialysis.

## 1 Introduction

Chronic kidney disease (CKD) is a major health problem worldwide. Characterized by the gradual loss of kidney function over time, CKD can progress to end-stage kidney failure,^[1]^ categorized as stage 5. These patients exhibit irreversible loss of kidney function, resulting in the accumulation of wastes and fluid, electrolyte imbalance, and acid alkaline imbalance. The progression of CKD is monitored by assessing several parameters. Decreased kidney size results from the loss of nephrons due to tubular atrophy, a hallmark of CKD.^[2]^ Glomerular dysfunction in CKD decreases the estimated glomerular filtration rate, resulting in increased concentrations of serum creatinine from muscle metabolism and blood urea nitrogen (BUN), a normal waste product from the breakdown of proteins in food.

Once the kidneys have failed, the patient faces the choice of 3 management options: hemodialysis (HD), peritoneal dialysis, or transplantation. The decision to transplant is made carefully. This surgical procedure itself is taxing on a patient already ill, one likely suffering from comorbidities such as hypertension or diabetes.^[3]^ Patients on maintenance dialysis experience an overall burden of physical and emotional symptoms, depression, and low quality of life.^[4]^ In addition, many patients with end-stage renal disease are elderly. In patients over 75 years of age, approximately 40% are affected by CKD, with a 1-year mortality rate following the initiation of dialysis of 41%.^[5]^ Therefore, the identification of potential palliative treatments for patients with CKD and end-stage renal disease is needed.

Active compounds have been identified in many plants used in Chinese medicine, with a broad range of important biological activities reported. For example, *Ligustrum lucidum* and its constituents are reported to exhibit hepatoprotective effects, anticancer activity, antioxidant activity, immunomodulating effects, antiviral activity, and antiosteoporosis activity.^[6]^ Compounds in *Rhodiola sacra* were observed to reduce pulmonary hypertension.^[7]^*Astragalus*, a dried root known as *Huang Qi* in Chinese, has demonstrated a wide range of immunopotentiating effects and has proven efficacious as an adjunct cancer therapy.^[8]^*Panax quinquefolius*, commonly known as ginseng, possesses anticancer, antidiabetic, and immunomodulatory activities and has been shown to improve the central nervous system function.^[9]^*Codonopsis pilosula*, a flowering plant also known as dang shen, has demonstrated effects against tumors, diabetes, and aging and has positive effects on the gastric mucosa, blood system, and immunity.^[10]^

We herein described the case of a 79-year-old woman with stage 5 CKD who chose not to undergo HD despite a rapid elevation in serum creatinine concentration from 3.85 to 7.25 mg/dL within 9 months. This deterioration of kidney function was palliated by the administration of Eefooton, a liquid formula of herbal extracts containing *Astragalus membranaceus*, *C pilosula*, *L lucidum*, *P quinquefolius*, and *R sacra*.

## 2 Case report

A 79-year-old woman with gout had diagnosed with stage 5 CKD which was characterized by renal impairment, primary hypertension, and chronic nephritic syndrome with unspecified morphologic changes and presented to the clinic on March 2016.

**Figure 1:**
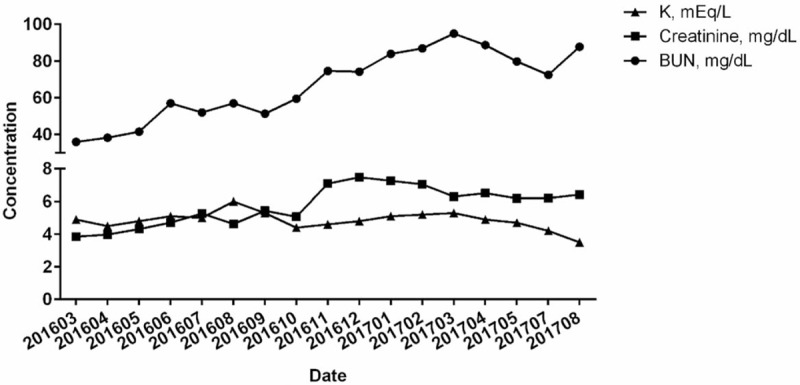
Trends of BUN, creatinine, and K concentrations before the Eefooton treatment. The patient had received the biochemical blood examinations every month before the Eefooton treatment from March 2016 to August 2017 in a total of 2 years. BUN = blood urea nitrogen, K = potassium.

During a 2-years observation period, the trend of serum creatinine concentrations of the patient was increased dramatically within 9 months (from March to December 2016) from 3.85 to 7.25 mg/dL (Fig. 1). The patient was also treated for hypoglycemia, hypertension, and heart disease by her physician. Her regular daily medications were included lansoprazole 30 mg 1 a day (QD), colchicine 0.5 mg QD, calcium carbonate 500 mg 4 times each day (QID), folic acid 5 mg QD, Hi-Beston 50 mg QD, ferrous gluconate B 300 mg + Vit B1 10 mg + Vit C 30 mg twice a day (BID), febuxostat 80 mg/tab 0.5 every other day (QOD), losartan potassium (K) 50 mg every night (QN), MPEG-Epoetin beta 100 μg (0.3 mL) every morning (QM), sodium bicarbonate 600 mg BID, and sennoside A + B calcium 12 mg take at bedtime (HS). During the intervention, the patient did not receive dialysis from any other clinics or hospitals.

After the outpatient treatment and examinations, she had started to take 20 mL of Eefooton BID on August 29th, 2017 for a total of 6 months. Eefooton is a liquid formula of herbal extracts consisted of *A membranaceus* 3 g, *C pilosula* 3 g, *L lucidum* 3 g, *P quinquefolius* 1.3 g, and *R sacra* 1.3 g in 20 mL water, and has the ISO22000 and hazard analysis and critical control points certifications approved by United Kingdom Accreditation Service. In addition to receiving Eefooton treatment, she continued taking her regular medications with a minor change including lansoprazole and losartan K were withdrawn; carvedilol 25 mg/tab QD, esomeprazole MUPS 40 mg QD, and Ultracet 1 tab PRN were added. Due to the desired effects of the herbal formula had achieved, Eefooton was stopped in March 27th, 2018.

**Figure 2:**
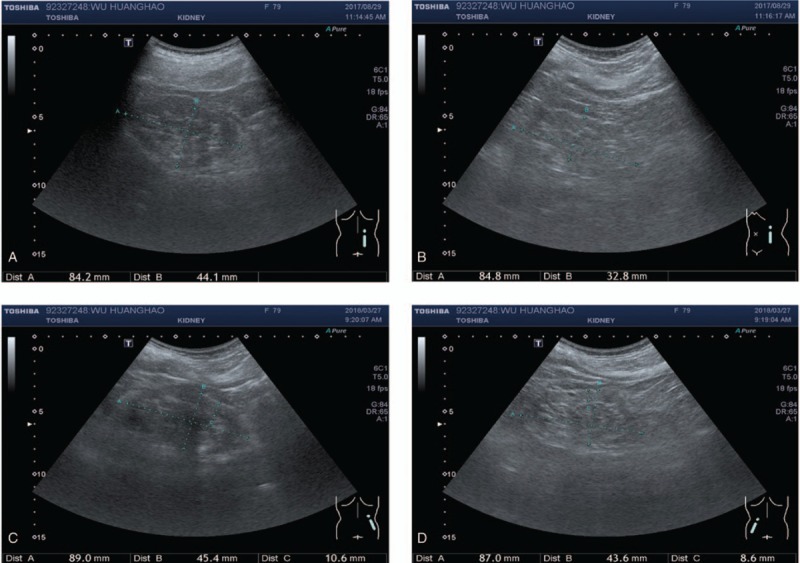
Ultrasonography scanning at outpatient clinic before and after Eefooton treatment. The patient had received the ultrasound scanning of both sides of kidney at outpatient clinic before the Eefooton treatment on August 29th 2017 (right: A, left: B) and at the end of 6-month Eefooton treatment on March 27th 2018 (right: C, left: D).

The therapeutic effect of Eefooton treatment was positive. Before the Eefooton treatment (on August 29th 2017), the ultrasonography of the patient had shown that the size of the right kidney was measured at 8.42 × 4.41 cm^2^ and that of the left kidney was at 8.48 × 3.28 cm^2^ (Fig. 2A and B). After the Eefooton treatment (on March 27th 2018), the ultrasonography had revealed that both kidneys had increased in size (8%), with the right kidney measuring at 8.90 × 4.54 cm^2^ and the left 1 at 8.70 × 4.36 cm^2^ (Fig. 2C and D).

**Figure 3:**
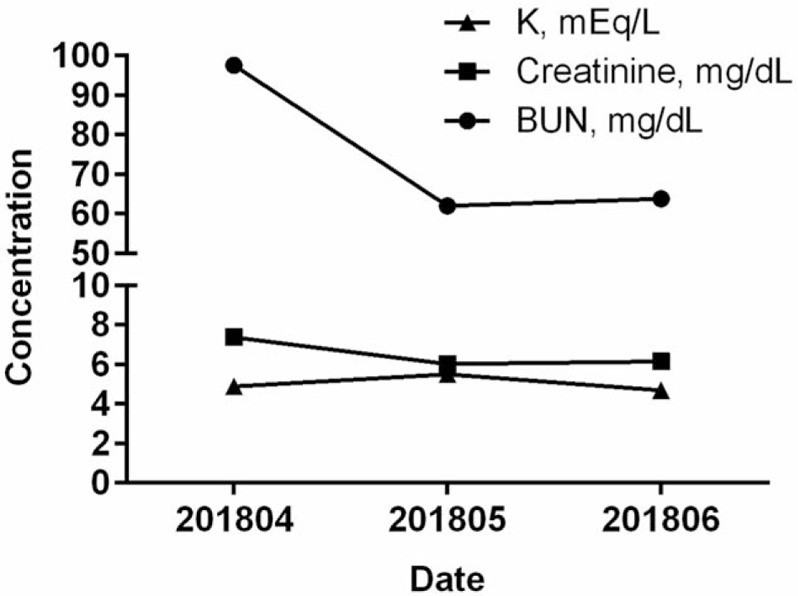
Trends of BUN, creatinine, and K concentrations after the end of Eefooton treatment. After the end of Eefooton treatment on March 2018, the biochemical blood examinations in the patient were followed up for 3 months, on April 24th, May 22th, and June 19th 2018. BUN concentration further decreased at 2 months after the completion of Eefooton treatment. BUN = blood urea nitrogen, K = potassium.

As was shown in Table 1, blood biochemical parameters of the patients before and after the Eefooton treatment were presented. After the Eefooton treatment, we had observed that there were elevated levels of hemoglobin, hematocrit, BUN, calcium, and CO_2_ in the patient's blood, while the concentrations of sugar, creatinine, inorganic phosphate, and K were decreased. In addition, the BUN and creatinine concentrations were further reduced at 2 months follow-up after the completion of the Eefooton treatment and remained steady (Fig. 3).

**Table 1:**
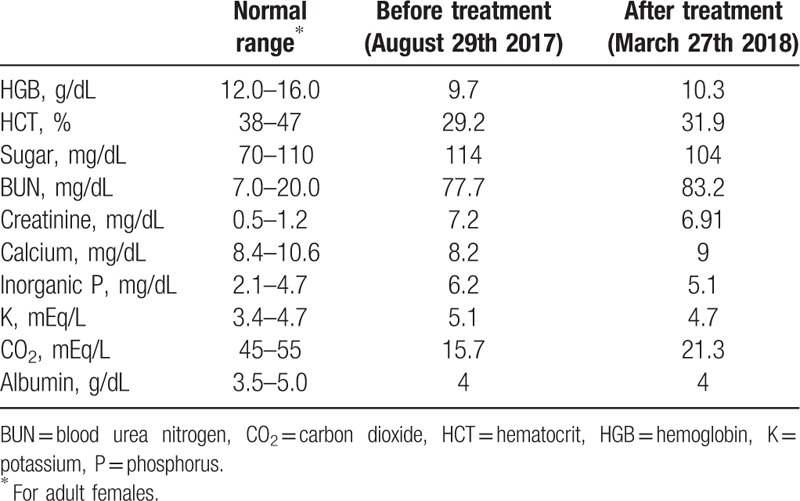
Blood biochemical parameters of the patient before and after the Eefooton treatment.

## 3 Discussion/conclusion

Here, we had reported the improvements in kidney function with the Eefooton treatment in a patient with stage 5 CKD who had decided against dialysis. Concerned about her rapid progression of the kidney atrophy, we had enquired that the size of each kidney in the patient should be monitored by her physician during the 2-years observation period. After the 6-months Eefooton treatment, the sizes of both kidneys in the patient were increased by about 8% (right, 8.42 × 4.41–8.90 × 4.54 cm^2^; left, 8.48 × 3.28–8.70 × 4.36 cm^2^, respectively) with no sign of factors causing the pathological kidney enlargement, such as tumors, kidney stones, and hydronephrosis. The trend of rising serum creatinine concentration was abated at the end of the 6-months Eefooton adjunctive treatment and remained at the elevated level but not continuously increased. These observations had suggested that her renal atrophy had been slightly recovered. Therefore, the administration of Eefooton had slowed the progression of CKD in the patient.

Although the Eefooton with the unique herbal formulation has not been explored previously, the effects of herbal extracts in the Eefooton liquid formula on the kidney function have been reported. Eefooton is a combination of herbal extracts included *A membranaceus*, *C pilosula*, *L lucidum*, *P quinquefolius*, and *R sacra*. *A membranaceus* has been commonly used as a hepatoprotective drug.^[11,12]^ In a study of 86 patients with CKD, *A membranaceus* treatment for 3 months was observed to significantly decrease serum creatinine levels in patients,^[13]^ indicating improved kidney function. A review of studies on the effects of *A membranaceus* on kidney function had reported that it had significantly reduced the serum creatinine especially in patients with baseline serum creatinine level at >133 μmol/L,^[14]^ or 1.52 mg/dL. The serum creatinine concentration of our patient was 7.2 mg/dL before the Eefooton treatment and decreased after the treatment, which is consistent with these reports. Although allergic reactions were the most commonly reported adverse events; no adverse effects were observed in *A membranaceus* treatment.^[14]^*L lucidum*, another component in Eefooton, exhibits antiosteoporotic effects and has the ability of improving kidney deficiency.^[15]^ An increase in serum calcium was observed by *L lucidum* ethanol extract treatment, possibly through the upregulating the transcriptions of calcitropic genes in kidney.^[16]^ At the end of the Eefooton treatment, our patient had an increase in serum calcium concentration, rising from abnormally low into the normal range (8.5–10.2 mg/dL). Elevated BUN and serum creatinine levels in kidney-damaged mice were reduced by the treatment with *P quinquefolius*.^[17]^ In our case, the patient had experienced a decrease in BUN level after the Eefooton treatment. *C pilosula* modulates immunomodulation and inflammation which increase granulocyte macrophage colony-stimulating factor secretion by macrophages and thus may enhance hematopoiesis in the setting of CKD.^[18,19]^ Despite its antioxidant effects that reduced tissue injury, *R sacra* may alleviate the renal damage.^[20]^ Thus, the improvements in kidney function observed in our study may be in accordance with previous reports of Eefooton individual components. There may be a possible synergistic effect of herbal extracts in Eefooton for our case.

After the administration of Eefooton, the improved kidney function was observed in our patient. No previous case report focused on the use of Eefooton for CKD. Eefooton may have an inhibitory effect on BUN and serum creatinine levels which could improve the glomerular filtration rate and prevent the kidney atrophy by reversing the loss of glomerular cells in CKD. The use of Eefooton for CKD may be beneficial for patients with abnormal serum creatinine level approximately up to 7 mg/dL, or patients require the imminent dialysis initiation. However, the present study was a preliminary clinical observation of 1 patient. Eefooton was used as an adjuvant treatment in addition to Western medicines, the observed improvement of kidney function in the patient attributed as the sole effect of Eefooton could not be determined. The long-term effect, prognosis, the possible mechanism of Eefooton would require further investigations.

It is worth noting that we are planning a well-organized clinical trial as a cohort study in the efficiency of Eefooton on the end-stage CKD patients.

The treatment with invasive HD or peritoneal dialysis is compulsory for the survival of the end-stage CKD patients. In our case, we shared our experience with Eefooton, highlighting its positive effect on a stage 5 CKD patient with no dialysis. Our result had revealed a potential treatment option for the end-stage patients who refuse these invasive procedures.

## Author contributions

**Conceptualization:** Chien-An Yao.

**Data curation:** Chih-Hui Lin.

**Formal analysis:** Chien-An Yao, Chih-Hui Lin.

**Investigation:** Chien-An Yao.

**Writing – original draft:** Chien-An Yao, Chih-Hui Lin.

**Writing – review & editing:** Chien-An Yao, Chih-Hui Lin.
